# Green Total-Factor Energy Efficiency of Construction Industry and Its Driving Factors: Spatial-Temporal Heterogeneity of Yangtze River Economic Belt in China

**DOI:** 10.3390/ijerph19169972

**Published:** 2022-08-12

**Authors:** Dalai Ma, Na Zhao, Fengtai Zhang, Yaping Xiao, Zuman Guo, Chunlan Liu

**Affiliations:** 1 School of Management, Chongqing University of Technology, Chongqing 400054, China; 2 Rural Revitalization and Regional High-Quality Development Research Center, Chongqing University of Technology, Chongqing 400054, China; 3 School of Economics, Chongqing Finance and Economics College, Chongqing 401320, China

**Keywords:** green total-factor energy efficiency of construction industry (CIGTFEE), Super-EBM, spatial-temporal heterogeneity, driving factors, the Yangtze River Economic Belt (YREB)

## Abstract

With the proposal of the “carbon peak, carbon neutral“ goal, energy efficiency has become one of the key means to achieve energy conservation and emission reduction at this stage. The construction industry, as a cornerstone of China’s economy, is characterized by serious overcapacity, energy waste, and pollution. As a result, academic research on its energy efficiency is gaining traction. This paper employed the Super-EBM model considering undesirable output to evaluate the green total-factor energy efficiency of the construction industry (CIGTFEE) in the Yangtze River Economic Belt (YREB) from 2003 to 2018. The spatial-temporal evolution characteristics and spatial heterogeneity of CIGTFEE were analyzed in detail through geospatial analysis. Finally, the driving factors of CIGTFEE were analyzed through a spatial econometric model. The results indicated that, during the sample research period, the CIGTFEE showed a holistic growth trend with volatility. By region, the downstream CIGTFEE grew sharply until 2006 and then remained fairly stable, while the midstream conformed to the “M” trend and the upstream region showed an inverted u-shaped trend; From the perspective of spatial differentiation, the CIGTFEE in YREB shows a significant spatial agglomeration situation, while the spatial agglomeration degree weakened. It existed a ladder-shaped change trend, with the regional average CIGTFEE from high to low levels as follows: Downstream, Midstream, and Upstream, and showed an obvious polarization in the upstream and downstream. From the analysis of the driving factors, CIGTFEE is significantly promoted by economic growth, energy structure, and human capital and suppressed by urbanization level, yet the impact of technological progress and the level of technology and equipment is not significant. In summary, province-specific policies based on spatial and temporal heterogeneity were proposed to improve the CIGTFEE of YREB.

## 1. Introduction

With regard to global warming, China, as the largest developing country and the largest coal consumer in the world, put forward in time the “double-carbon” goal, i.e., the “carbon peak, carbon neutral” goal, and the goal of national independent contribution to climate change, so as to reduce energy consumption per unit GDP by 13.5% and CO_2_ emissions by 18%. As the demonstration belt of ecological civilization construction, the Yangtze River Economic Belt is the main force to achieve the above-mentioned goals. The rapid economic development and the continuous improvement of urbanization levels in the Yangtze River Economic Belt depend on traditional energy, and the problems of resource depletion and environmental degradation have already emerged. However, the International Energy Agency (IEA) predicts that it will take 15–20 years for China’s industries to achieve deep decarbonization, so the short-term benefits of improving energy efficiency are far greater than those of energy decarbonization, and it is an important measure to promote high-quality economic development and protect the environment at a high level. Therefore, improving energy efficiency is still one of the main means to achieve energy conservation and emission reduction at this stage.

Construction, as one of the pillar industries in China, greatly promoted the development of the Chinese economy. However, as a resource-intensive industry, construction realizes its development at the cost of consuming a huge amount of energy. Consequently, improving the energy efficiency of the construction industry is conductive to reducing CO_2_ emissions, alleviating environmental pollution, and finally realizing the “double carbon ” goal.

Furthermore, the difference in technological competitiveness and economic development levels of each province in YREB results in unbalanced development and differentiated distribution in the energy efficiency of the construction industry. Thus, it is essential to study the spatial-temporal evolution characteristics of energy efficiency in YREB’s construction sector. Exploring the energy efficiency of various provinces can contribute to better understanding YREB’s energy utilization status and provide policy implications for improving YREB’s energy utilization efficiency [1].

However, existing studies have rarely focused on the regional differences and the spatial analysis. So, this paper is committed to making the following contributions: First, to scientifically and objectively estimate the CIGTFEE of YREB. This paper measures CIGTFEE considering bad output from an input-output perspective. Which enriched the research on evaluation of the efficiency of energy resources exploitation in the YREB; Second, exploring its spatial-temporal heterogeneity from a multidimensional perspective. This article fills the research gap in the temporal-spatial differences between regional CIGTFEE; Third, identifying the relevant driving forces. This paper fills up the research gap on the factors driving the spatiotemporal dynamics of CIGTFEE. Considering the spatial dependence between observations, a spatial econometric model was established to identify these driving factors. The results obtained through this model are more consistent with the actual results of YREB and better than non-spatial econometric model methods, such as the ordinary least squares (OLS) model or Tobit model.

The rest of this paper is organized as follows. Section 2 reviews and combs the related literature. Section 3 introduces the method, indicators, and datasets used in this paper. The measured results, spatiotemporal dynamics, spatial correlations, and spatial econometric regression, are described and discussed in Section 4. The results are summarized, and policy implications are given in the last section.

## 2. Literature Review

With the rapid development of the economy and the acceleration of urbanization in China, the construction sector has grown into the main energy consumer and pollutant emitter. The energy efficiency of the construction industry has attracted much attention from the government and academia. Promoting energy efficiency in the construction industry is the key to alleviating resource, energy, and environmental constraints, improving development quality, and achieving the goal of energy saving and emission reduction [2]. Therefore, an effective evaluation of the energy efficiency of the construction industry is necessary.

In terms of existing research of the energy efficiency issue, single-factor energy efficiency (SFEE) and total-factor energy efficiency (TFEE) were the two major categories [3]. SFEE has two main shortcomings, one is that it only considers the impact of energy inputs on production output, ignoring the substitution of other factors of production for energy [4]; the other is that it does not reflect the role of technological progress. Thus, SFEE cannot comprehensively evaluate the entire production system’s energy efficiency. To solve the defects, the TFEE concept [5] and data envelope analysis (DEA) [6] were proposed, in which, DEA, as a non-parametric analytical approach, utilizes mathematical programming to solve the relative efficiency problem of decision-making units (DMUs) with multi-inputs and multi-outputs. This model is also characterized by many advantages. For instance, it can be used to evaluate the efficiency value with no need to make a priori assumptions on the underlying functional form and information on prices. Therefore, when measuring TFEE, the DEA model is the most popular calculating tool that scholars choose. The DEA model has been widely applied to evaluate the energy efficiency or environmental performance assessment of many sectors, such as industrial sectors [7,8,9], agricultural sectors [10,11], energy-intensive industries [3], and so on. However, the traditional DEA model has two concerns: the disposal of undesirable outputs and the impact of external factors and random error, which promoted the evolution of the DEA model [2]. Tone extended the traditional DEA method and put forward a non-radial, non-angle SBM model, which perfectly solved the problem of undesired output [12]. Chen [2] and Zhang [13] applied a three-stage undesirable SBM-DEA model to measure the energy efficiency in the construction industry of China. Subsequently, Tone improved the SBM model to the Super-SBM model because it further solved the problem of cannot ranking DMUs whose efficiency are all one [14]. The Super-SBM model is widely applied to estimate the energy efficiency of the construction industry [15,16]. However, the Super-SBM model, as a derivative method of SBM, had the same deficiency: not taking into account the radial and non-radial compatibility of the input-output variables, causing the low efficiency of the evaluation decision unit. To overcome the defects of Super-SBM, Tone [17] further improved the SBM model into the EBM model. Yang evaluated the regional ecological energy efficiency for 30 regions in China and its three major areas for the period 2007–2015, applying an EBM model [18]. Similarly, the Super-EBM model, as a further optimization of the EBM model, fully integrates EBM with super-efficiency DEA [7]. Compared with traditional DEA model and the Super-SBM model, the Super-EBM model can evaluate industrial energy efficiency scientifically. Nonetheless, few scholars have applied this model to evaluate the energy efficiency of the construction industry. To provide objective results for efficiency comparison, the Super-EBM model would be adopted to evaluate the CIGTFEE of YREB’s 11 provinces in this paper.

The regional spatial structure is the key to sustainable development of the YREB [19]. The differences in natural resources, geographic location, and market environment in each region of the YREB have led to uneven development of the regional construction industry and an ensuing substantial disparity in regional energy utilization and CIGTFEE. Thus, regional differences in energy efficiency have been studied by some scholars. Xue [20] estimated the total energy consumption efficiency in construction industry and found that energy conservation gaps exist between the northeastern, western regions and the central, eastern regions. The results of several studies also indicate that energy efficiency in the building sector has spatial agglomeration and spatial-temporal heterogeneity [21,22]. However, prior studies on the CIGTFEE have not considered the two major geographical characteristics of spatial correlation and regional differences. Xu [23] believed that only understanding regional distribution and its evolution, can we develop distinctive and effective strategies for improving energy efficiency for different provinces. Consequently, studying the evolution characteristics of spatial and temporal distribution in the CIGTFEE will not only reveal changes in the distribution of CIGTFEE, but will also enable further exploration of the causes of regional differences and ultimately provide a basis for the development of regional carbon reduction policies.

The current studies of the driving forces on energy efficiency of construction industry have focused on different scales and methods. Some scholars have investigated how energy poverty [24], technological progress [25], environmental regulation [13,26] and urbanization [27] exert the influence on energy efficiency of construction industry in China. Some discussed composite factors that influence energy efficiency of construction industry. For example, Wang [28] utilized production-theoretical decomposition method to explore the driving factors of energy-related CO_2_ emissions (ECE) from the construction industry, including industrial activity; advances in industrial output technology; the effects of potential energy intensity changes; industrial output technical efficiency; changes in spatial structure;energy-saving technology; and energy consumption structure and energy usage efficiency. Liang [29] used a stochastic frontier method and constructed an external evaluation system from the four aspects of economic growth, technological development, policy support, and industrial development level, and found that construction industry energy efficiency is promoted by GDP per capita, energy consumption structures, industrial development degrees, and industrial concentrations, and inhibited by urbanization levels, technical equipment, policy support, and marketization. All these empirical analysis aboutinfluencing factors, carried out most are based on non-spatial econometric models. These models might have serious bias in estimation because of their failure to consider the potential spatial correlations between observations. The spatial econometric model making the analysis results of influencing factors more in line with reality. Therefore, the spatial econometric model has been favored by more and more scholars. For example, Xie employed the spatial econometric model to empirically measure the drivers of energy efficiency of construction industry (CIEE) and reveal that CIEE is significantly promoted by enterprise scale, property right structure, and environmental regulation, greatly inhibited by economic growth and technical level, and not greatly affected by production level or urbanization level [15]. However, there is still little research on applying the spatial econometric model to energy efficiencyof construction industry.

Based on the above, existing research mostly took China as the research object and explored the energy efficiency of the construction industry without considering undesirable output and spatial-temporal evolution. In addition, empirical analysis of driving factors not considered the potential spatial correlations between observations. To solve these problems, this study intends to carry out related research work from two aspects. (1) this study aims to measure the CIGTFEE of 11 provinces in YREB, China, from 2003 to 2018, by employing Super-EBM model with full consideration of bad outputs and exploring the spatial and temporal characteristics of CIGTFEE in YREB. (2) From the perspective of spatial heterogeneity, this study uses a spatial econometric model to reveal the driving factors of CIGTFEE in the Yangtze River Economic Belt. Then, the study proposes countermeasures and suggestions.

## 3. Materials and Methods

### 3.1. Study Area

**Figure 1:**
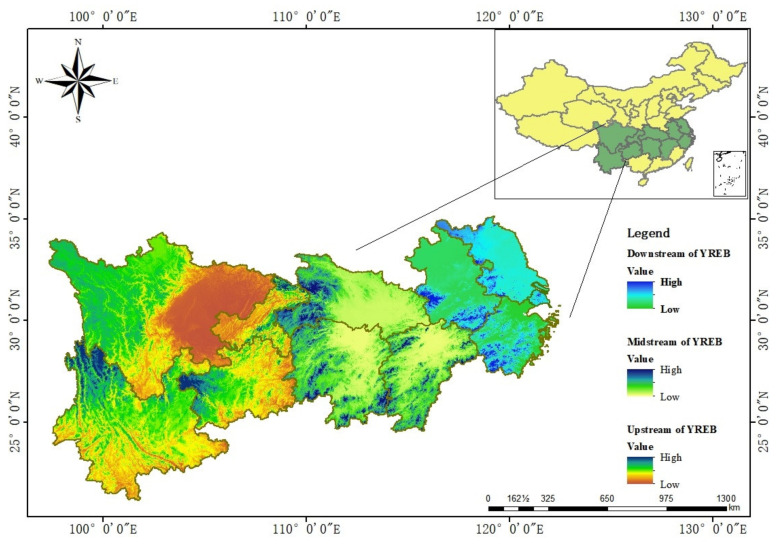
The location map of the study area.

As one of the “three major strategies” implemented by the central government, the Yangtze River Economic Belt (YREB) passes through the eastern, central, and western plates, covering Shanghai, Jiangsu, Zhejiang, Anhui, Jiangxi, Hubei, Hunan, Chongqing, Sichuan, Guizhou, and Yunnan. Relying on the resource advantages of the Yangtze River Golden Waterway, the YREB has achieved rapid economic development and become the main force driving China’s high-quality economic development. It accounts for about 40% of the country’s total economy, and it is in a relatively strong position. It is an inland river economic belt with global influence. However, serious environmental problems arise with the development of the YREB. The environment is relatively fragile, and the ecological development of industry has a long way to go. There is still room for improvement in the development of the construction industry. The location map of the study area is shown in Figure 1.

### 3.2. Methods

**Figure 2:**
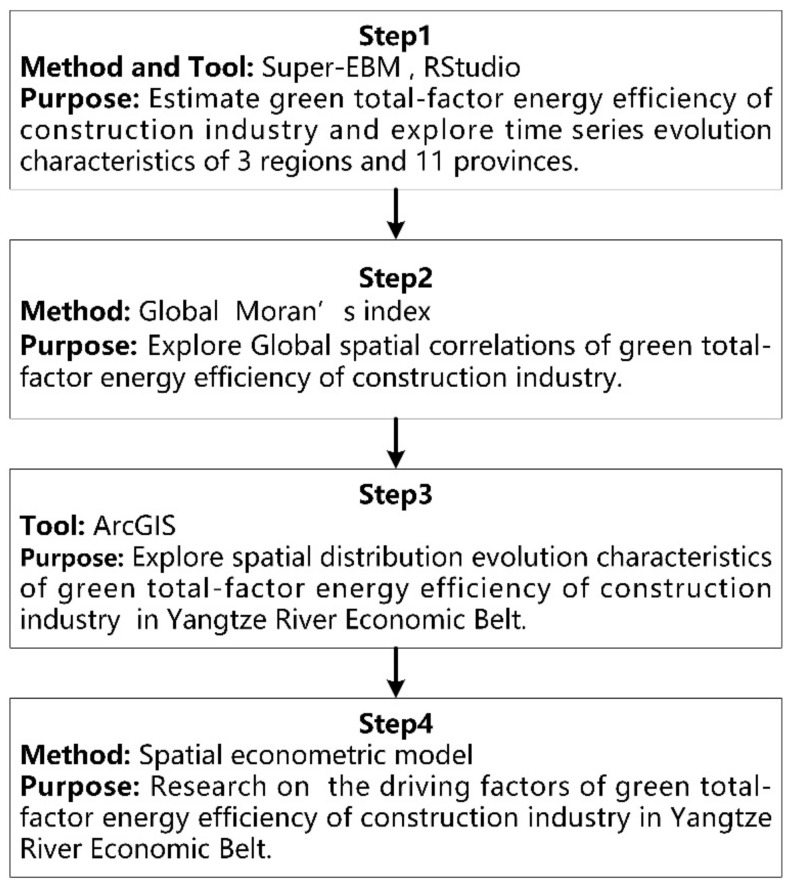
Overview of this study.

To truly and objectively estimate CIGTFEE in YREB, Super-EBM was used in this study. The spatial-temporal differences were visualized by applying R Studio and ArcGIS software to the results of Super-EBM model. In addition, a spatial econometric model was implemented to explore driving factors of CIGTFEE. The method and tool used in this paper were structured as shown in Figure 2.

#### 3.2.1. The Super-EBM Model Based on Undesirable Output

Radial DEA models, such as BCC and CCR (named after Charnes, Cooper, and Rhodes), are widely used to measure energy efficiency. However, these models take the maximization of output as the precondition, which not only fails to take into account the non-radial slack variables but also fails to include undesirable outputs such as environmental pollution, which often leads to bias in the evaluation results. To solve these problems, Tone proposed a non-radial and non-angle SBM model, which perfectly solves the problem of undesirable output. However, the SBM model did not consider the compatibility of radial and non-radial input-output variables at the beginning of its establishment, which resulted in the evaluation of DMU being lower than it actually was. Therefore, Tone and Tsutsui further improved the SBM model into the EBM model, which is perfectly compatible with the radial and non-radial relationships of input-output variables. However, when the efficiency values of multiple DMUs are all one, it is impossible to effectively distinguish the efficiency of their DMUs. At this time, the appearance of the Super-EBM model is a further optimization of the EBM model. Its biggest advantage is that it breaks through the restriction that the maximum efficiency value of DMUs is one, and can more accurately and effectively sort multiple DMUs with an efficiency value of one.

According to Tone [14], this paper constructs a production system composed of n DMUs. Each DMU has m production factors as inputs $X=(x_{1},x_{2},\ldots ,x_{n})\in R_{+}^{m\times n}$, and produces $s_{1}$ desirable output $Y=(y_{1},y_{2},\ldots ,y_{n}^{})\in R_{+}^{s_{1}\times n}$, $s_{2}$ undesirable output $B=(b_{1},b_{2},\ldots ,b_{n})\in R_{+}^{s_{2}\times n}$ separately. $P^{t}(x)=\{(x,y,b):x\;\mathrm{can produce}\;y\;\mathrm{and}\;b\}$ indicates all possible production sets produced. The decision unit *j* to be estimated is $DUM_{j}=(x_{j},y_{j}^{},y_{j}^{})$. The Super-EBM model containing the undesirable output is as follows:

(1) $$\begin{array}{l} \eta^{*}=\min\frac{\gamma +\sigma_{x}\sum_{i=1}^{m}\frac{w_{i}^{-}s_{i}^{-}}{x_{ij}}}{\omega -\sigma_{y}\sum_{p=1}^{s_{1}}\frac{w_{p}^{+}s_{p}^{+}}{y_{pj}}+\sigma_{b}\sum_{q=1}^{s_{2}}\frac{w_{q}^{b-}s_{q}^{b-}}{b_{qj}}}\\ s_{.}t_{.}\;\;\;\gamma x_{ij}\geq \sum_{j=1}^{n}x_{ij}\lambda_{j}-s_{i}^{-},i=1,\ldots ,m\\ \;\;\;\;\;\;\;\;\;\;\;\;\omega y_{pj}\leq \sum_{j=1}^{n}y_{pj}\lambda_{j}+s_{p}^{+},p=1,\ldots ,s_{1}\\ \;\;\;\;\;\;\;\;\;\;\;\;\;\;\omega b_{qj}\geq \sum_{j=1}^{n}b_{qj}\lambda_{j}-s_{q}^{b-},q=1,\ldots ,s_{2}\\ \;\;\;\;\;\;\;\;\;\;\;\;\;\lambda \geq 0,\;\;\;s_{i}^{-},s_{p}^{+},s_{q}^{b-}\geq 0 \end{array}$$

where $\eta^{*}$ represents the score of CIGTFEE of Super-EBM; $\lambda_{j}$ is linear combination coefficient of *j*-th DMU, reflects the relative importance of *j*-th DMU; *x*, *y*, and *b* respectively represent the input, desirable output, and undesirable output, therefore, $x_{ij}$, $y_{pj}$, $b_{qj}$ corresponds to the *i*-th input, *p*-th good output and *q*-th bad output of the *j*-th DMU, respectively; $w_{i}^{-}$, $w_{p}^{+}$ and $w_{q}^{-}$ are the weights of input, good output and bad output, respectively; γ and ω are the planning parameter of the radial part; $\sigma_{x}$, $\sigma_{y}$ and $\sigma_{b}$ respectively are the non-radial weights of input, good output and bad output, respectively, and they are limited to [0, 1]; $s_{i}^{-}$, $s_{p}^{+}$ and $s_{q}^{b-}$ are the slack term of input, good output and bad output, respectively.

#### 3.2.2. Moran Index

Verifying the existence of spatial correlation of total factor energy efficiency of the construction industry in YREB is an important question to be studied in this paper. Spatial correlation, which can also be called spatial dependence, refers to the observation data reflecting some variables, which may not be spatially independent of each other because of the influence of spatial interaction and spatial diffusion. Generally speaking, the global Moran’s I index is used as a measure of spatial correlation. The formula is as follows:

(2) $$\mathrm{Moran}’sI=\frac{n}{\sum_{i=1}^{n}{\left(x_{i}-\bar{x}\right)}^{2}}\frac{\sum_{i=1}^{n}\sum_{j=1}^{n}W_{ij}(x_{i}-\bar{x})(x_{j}-\bar{x})}{\sum_{i=1}^{n}\sum_{j=1}^{n}W_{ij}}$$

In Equation (2), *n* is the number of target provinces along YREB; $x_{i}$ and $x_{j}$ are the observations of provinces *i* and *j*, respectively; $\bar{x}=(\sum_{i}x_{i})/n$ reflects the mean observation of all provinces; The value range of Global Moran’s I is [−1, 1]. If the index surpass 0, and passes a certain significant level test, this indicates a significant positive spatial correlation among the observed provinces; If the Moran’s I index is less than 0 and passes the significant level test, it means a negative spatial correlation among the observed provinces; If, and only if, the index is 0, the provincial observations are independent in space; $W_{ij}$ is adjacency matrix, to facilitate the characterization, the Rook adjacent space weight matrix composed of 0 and 1 is used, the specific expression forms are as follows:

(3) $$W_{ij}=\left\{\begin{array}{l} 1\;\;\;\;\;\;\;\;\;\;\;province\;i\;\mathrm{and}\;j\;\mathrm{are}\;\mathrm{adjacent}\\ 0\;\;\;\;\;\;\;\;\;\;\;province\;i\;\mathrm{and}\;j\;\mathrm{are}\;\mathrm{not}\;\mathrm{adjacent}\; \end{array}\right.$$

### 3.3. Construction of the Indicator System

The total factor energy efficiency of the construction industry reflects the ratio of the optimal energy consumption of the construction industry to the actual energy consumption of the construction industry when the economic output is optimal and environmental pollutants are minimized, on the premise that the inputs of capital, labor, and other production factors are unchanged. Based on the existing achievements and following the principles of systematicness, scientificity, and data availability, this paper establishes the evaluation index system of CIGTFEE in Table 1. The index system consists of input, good output, and bad output, in which the input includes three elements: energy, labor, and capital; the desirable output is the total output value of the construction industry; the undesirable output is expressed by the carbon emissions of the construction industry. The specific indicators are explained as follows:

(1) Energy input: The measurement of energy efficiency is the main objective of this paper. Energy input is the core input element of the whole index system. There are abundant types of energy consumption and a diversity of energy structures in the construction industry of each province. Energy consumption in the construction industry is mainly composed of 12 kinds of energy, such as coal, oil, natural gas, electric power, etc. If all kinds of energy were incorporated into the index system directly, it would not only be cumbersome but also the statistical caliber would be inconsistent. To unify and facilitate calculation, the total amount of the twelve types of energy consumption in the regional energy balance table is converted into standard coal according to the energy conversion coefficient of “10,000 t of standard coal,” and takes the calculated total energy consumption as an energy input.
(2) Labor input: The labor force is the main body of energy utilization in the construction industry. Only when energy is combined with labor force elements, can it really play its role. Generally speaking, labor hours are the best indicator to measure labor input, but considering that data on labor hours cannot be obtained directly, this paper selects “the number of employees in the construction industry” as the indicator to measure labor input.
(3) Capital input: According to the production function in economics, capital is the basic factor of production. When measuring the capital input, the perpetual inventory method (PIM) [30] is often used to estimate the capital stock in research. However, in view of the depreciation rate of fixed assets in the construction industry is not available, it is not feasible to directly estimate the capital stock of the construction industry as a capital input. Therefore, this paper directly adopts “investment in fixed assets of the construction industry” to measure capital input of the construction industry and uses the deflator method to convert it into actual investment in fixed assets of the construction industry, with 2003 as the base period.
(4) Economic output: The gross output of the construction industry, that is, the total value created by construction enterprises, can best reflect the total output level of the construction industry. On this account, this paper selects “total output of the construction industry” as the economic output. Similarly, to eliminate the influence of price factors, the current total output of the construction industry is transformed into the actual total output of construction in the region using GDP deflators with 2003 as the base period.
(5) Carbon dioxide emissions: At present, the development of the construction industry depends on the support of fossil fuel, but the utilization of fossil fuel will inevitably emit a large amount of direct and indirect emissions. Among them, carbon dioxide, as a typical representative of environmental pollutants, is the main control and emission reduction object in the energy utilization of the construction industry advocated by most scholars. It should be noted that because the relevant statistical yearbook has not yet given the direct data of carbon emissions from the construction industry, this paper estimates the energy carbon emissions by multiplying the physical quantities of various energy consumption in the regional energy balance tables in the China Energy Statistical Yearbook by the reference coefficients of each energy converted into standard coal in the General Principles for Comprehensive Energy Consumption Calculation, and then multiplied by the respective carbon emission coefficient, published in the IPCC (2006) document internationally.

### 3.4. Data Source

Based on the availability and comprehensiveness of data, this paper takes 11 provinces along YREB as the basic research unit, and the research period is from 2003 to 2018. All variables in this paper are from China Statistical Yearbook, China Labor Statistical Yearbook, China Energy Statistical Yearbook, and China Construction Industry Statistical Yearbook from 2004 to 2019. In addition, some missing data is filled in by the simple average method and the moving average method.

### 3.5. Driving Factors on CIGTFEE

To explore the factors driving CIGTFEE is another important objective of this research. Inspired by existing research, this paper selects Economic growth (EG), Technological progress (TP), Urbanization (UL), Energy structure (ES), Human capital (HC), Technology and equipment level (TL) serve as the driving factors of CIGTFEE. These factors are described as follows:

(1) Economic growth (EG): Previous studies have shown the relationship between energy consumption and economic growth is highly related to the actual social development in the study area [31,32]. EG is often accompanied by the agglomeration of talent, capital, and technology, and these factors are crucial to the development of the construction industry. However, the effects of EG on the ecosystem might be considerably more harmful. Thus, it is essential to investigate the correlation between EG and CIGTFEE. EG was measured by the per capita GDP of each province and city, and at the same time, in order to eliminate the influence of collinearity, the natural logarithm of this index was taken. Therefore, the nexus between EG and CIGTFEE is uncertain.
(2) Technological progress (TP): As a dominant contributor, TP is conductive to reducing energy consumption intensity [25,33]. Relevant research shows that TP improves the quality of energy inputs, significantly reduces the associated cost, frees up resources, and maximizes output [34]. Moreover, TP can upgrade the traditional high-energy equipment and improve the production efficiency of enterprises, and then promote the rational utilization of energy in the construction industry and reduce the energy consumption intensity, thus promoting the green transformation and growth of regional industries.TP is expressed by the ratio of regional R&D expenditure to GDP, and it is expected that TP promotes CIGTFEE.
(3) Urbanization level (UL): The level of urbanization is closely related to the energy efficiency of the building sector, and there are two contrasting views on the relationship between the two. One thing, urbanization is accompanied by population migration from rural to urban areas, stimulating housing demand and promoting the continuous expansion of the construction industry, which increases building energy consumption and emits a lot of carbon dioxide [27]. For another, the increasing level of urbanization may have facilitated the inflow of high-quality educational resources and skills, raising labor value, facilitating the development of energy-saving technology, and enhancing energy efficiency [29]. Therefore, the relationship between UL and CIGTFEE is uncertain. Here, UL is expressed by the ratio of the resident population in urban areas to the total population.
(4) Energy structure (ES): The optimization and adjusting of ES are conducive to slowing down energy consumption growth and are crucial to green construction development. Compared with traditional fossil fuels such as coal and oil, an increase in the proportion of electricity consumed could significantly enhance labor and energy efficiency [29]. The clean energy represented by electricity has higher energy conversion efficiency and lower carbon dioxide emissions. By increasing the ratio of electric energy consumption in the construction sector, it is possible to upgrade the ES and promote emission reduction in the construction sector. Therefore, ES can be expressed by the ratio of electric energy consumption to the total energy consumption in the construction industry and is expected to promote CIGTFEE.
(5) Human capital (HC): Scientific production skills, advanced production equipment, and well environmental consciousness are better mastered by high-quality employees, who can provide the necessary intellectual support for reducing energy consumption and promoting the green transformation of enterprises [35]. Therefore, this paper substitutes HC with the per capita years of education of the labor force in a region, and it is expected that HC promotes CIGTFEE.
(6) Technology and equipment level (TL): The increase in the ratio of technical equipment indicates an increase in investment in fixed capital [29]. This value is a measure of the level of technological development of the construction industry in terms of the value of fixed assets and equipment per person employed in the construction industry. The higher the technical equipment ratio in the construction industry, the higher the level of technology, and vice versa, the lower [36]. Therefore, it is expected that TL will promote CIGTFEE.

## 4. Results and Discussion

### 4.1. Regional Differences

According to Equations (1) and (2), Super-EBM was used to measure the CIGTFEE of 11 YREB’s provinces and cities from 2003 to 2018. The specific results are shown in Table 2.

**Figure 3:**
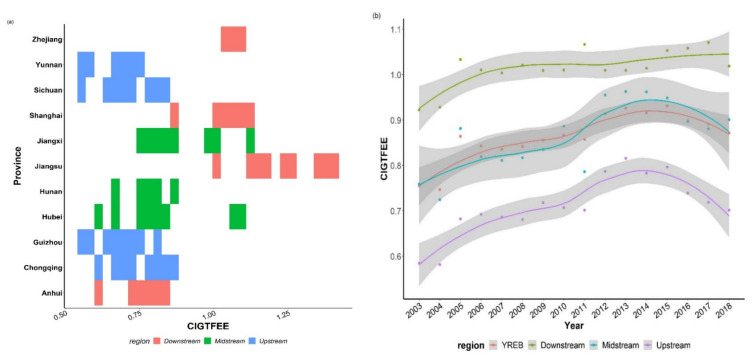
Regional and provincial level of the CIGTFEE in YREB. (**a**) shows the distribution of CIGTFEE at the provincial level. (**b**) displays the overall and three regions’ average CIGTFEE curves.

Figure 3a illustrates the provincial mean CIGTFEE across the period of observation, revealing significant provincial differences in CIGTFEE. At the provincial level (Figure 3a), the provinces in downstream, including Jiangsu, Zhejiang, and Shanghai, maintained high CIGTFEE levels. Their CIGTFEE are satisfactory due to two main factors: To begin with, these downstream regions are economically developed and pioneers in the construction sector. They have a higher level of building science and technology than the other provinces and cities of the YREB. Second, these provinces and cities aggressively executed the national green development policy and explored the road of green development: emphasizing the green and mechanized transformation of the construction industry, optimizing and updating construction industry, research and development of clean energy technologies and renovation of traditional construction equipment. These efforts effectively aided enterprise energy conservation and pollution reduction.

The provinces in the Upstream and Midstream sectors kept their CIGTFEE low. In the midstream and upstream of the YREB, developed provinces such as Chongqing, Hunan, and Hubei have higher CIGTFEE values than underdeveloped provinces such as Guizhou and Yunnan. Therefore, the economy facilitated the CIGTFEE. And relatively backward clean production technologies are undoubtedly another reason why Guizhou and Yunnan lag behind other provinces and cities in the upper and middle reaches.

From a regional level (Figure 3b), CIGTFEE were higher in downstream provinces than in midstream and upstream provinces. In YREB, the provincial differences in CIGTFEE exist and must be addressed to secure the overall effect of green construction transformation.

In Figure 3b, the CIGTFEE trends of YREB and those of downstream, midstream, and upstream regions are compared to further investigate regional differences in CIGTFEE. CIGTFEE in YREB was only 0.755 in 2003, but had climbed to 0.871 by 2018, representing a 15.36% growth in 16 years. Except for a brief spike in a few years, the CIGTFEE of downstream regions remained steady across the data period (2003–2005).

To further investigate the regional differences in the CIGTFEE, the CIGTFEE trends of the YREB and those of the downstream, midstream, and upstream regions are compared in Figure 3b. It can be seen that CIGTFEE during the research periods revealed an overall growing tendency. The CIGTFEE of the downstream regions remained stable during the sample period, except for a rapid grew in a few years (2003–2005). The CIGTFEE of the midstream regions exhibited an “M” shape with “two peaks and one valley”;The CIGTFEE in the upstream regions have an obvious inverted “U” curve. The CIGTFEE of the three regions differed markedly.

In summary, CIGTFEE was much higher in the downstream than in the YREB, upstream, and midstream zones. In the future, policymakers must pay more attention to regional variations in CIGTFEE and develop effective localized green transformation programs.

### 4.2. Spatial Correlations

#### 4.2.1. Global Spatial Correlations

Based on Equations (2) and (3), this paper calculated the average CIGTFEE of YREB in the four periods from 2003 to 2018 and used Geoda to evaluate the Moran’s index. The global auto-correlation results are given in Table 3.

As shown in Table 3, the CIGTFEE of YREB was always positive during four inspection periods, passing the significance level test, which fully shows that the CIGTFEE of YREB shows a significant positive spatial correlation feature. At the same time, the result also shows that CIGTFEE in neighboring provinces of YREB presents an obvious spatial agglomeration situation, that is, the provinces with higher or lower CIGTFEE are relatively concentrated in space, which indicates that the spatial distribution of CIGTFEE between regions is not random, and there is a strong imitation effect among neighboring provinces. In addition, over time, the global Moran index decreased, indicating the spatial agglomeration degree of CIGTFEE of YREB had weakened, but this spatial effect still had an important impact on the change of CIGTFEE. In a word, spatial correlation is an important driving factor that cannot be ignored in the empirical analysis of CIGTFEE of YREB, otherwise it will lead to a big deviation between the empirical results and the actual results.

#### 4.2.2. Spatial Distribution Evolution Characteristics

**Figure 4:**
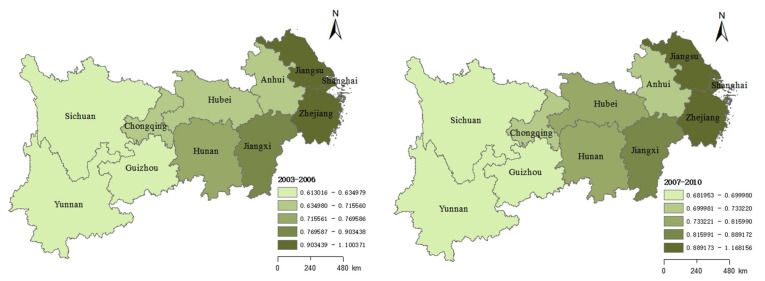

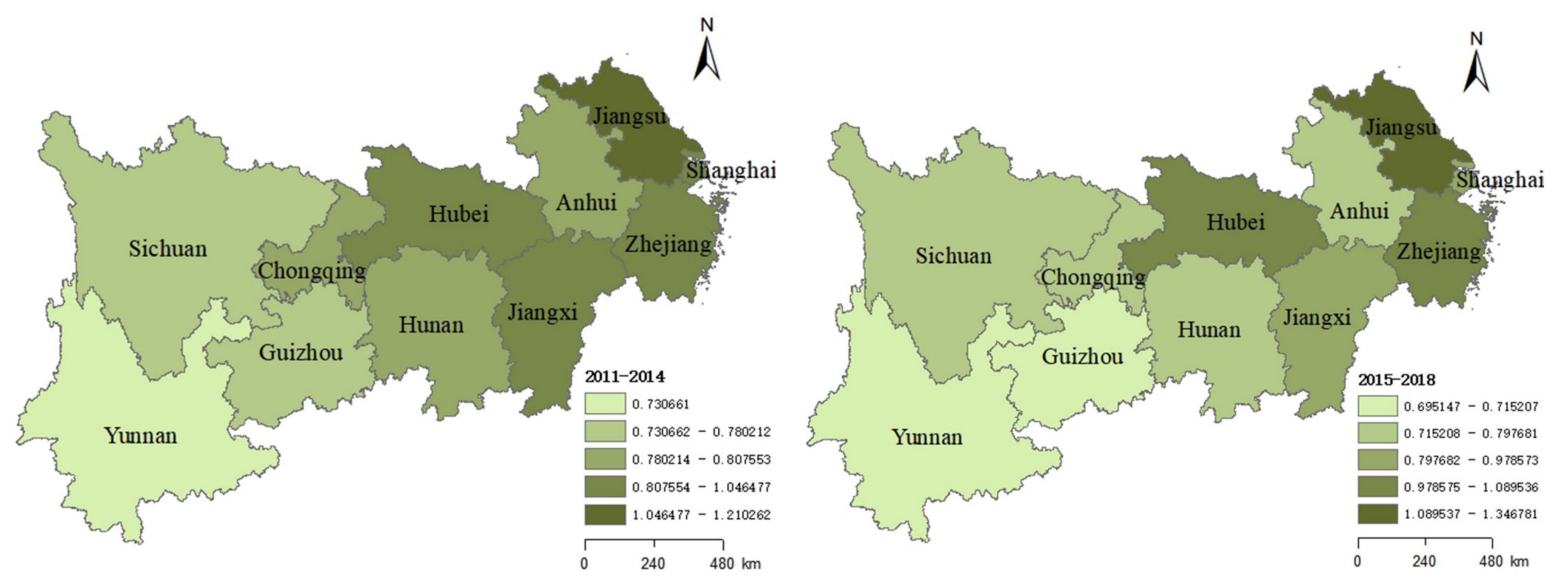
Changes of the CIGTFEE in YREB from 2003 to 2018.

To analyze the CIGTFEE, this study divided the CIGTFEE range into five equal parts by natural breaks, and referred to them as the lowest, low, medium, high, and highest levels, respectively. The ArcGIS 10.8 software was used to visualize the results of the CIGTFEE (Figure 4). The spatial situations of the regional and provincial level CIGTFEEs were interpreted as follows:

(1) As shown in Figure 4, the spatial distribution of CIGTFEE in YREB generally presents a ladder-shaped change trend, with the regional average CIGTFEE from high to low levels was as follows: Downstream, Midstream, and Upstream. This is directly tied to the variations in resource endowment and economic development between provinces. It can be seen that the provinces in the downstream and upstream regions show obvious polarization.
(2) During four periods, Jiangsu, Zhejiang were ranked in the highest efficiency range. Although the CIGTFEE in Shanghai showed a downward trend, it still placed high on the list.
(3) In contrast, Sichuan, Guizhou and Yunnan, which located in the economically and technologically underdeveloped upstream, always in a state of inefficiency. Compared with other upstream provinces, Chongqing’s CIGTFEE placed high during four periods. Therefore, in the upstream region, Chongqing should play a good role in leading the way, strengthen the cooperation between the upper, middle and lower reaches, and narrow the gap.
(4) CIGTFEE has to some extent been enhanced in Hubei. Moreover, CIGTFEE has declined to a certain extent in Hunan and Jiangxi.but Jiangxi still ranked first in the midstream.

Overall, the spatial spillover effect obviously exists in the YREB, CIGTFEE is comparable across nearby provinces and cities as a result of knowledge and technological spillover. This is fully reflected in Jiangxi and Chongqing. Spatial structure analysis offers a solid scientific foundation for enhancing regional development effectiveness and advancing regional sustainable development. Based on the above analysis, policymakers can rationally optimize and adjust the Yangtze River Economic Belt’s spatial structure so that the spatial interaction can reach its best state, maximizing the allocation of local resources and spatial synergy and hastening the region’s sustainable development.

### 4.3. Analysis of Driving Factors

Based on above analysis of provincial CIGTFEEs in YREB, there exists a significant temporal and spatial differentiation characteristics. The spatial econometric model performed better than the non-spatial regression model (OLS model) and Tobit model when dealing with the problem of spatial autocorrelation. That is, the spatial econometric model is suitable for the analysis on CIGTFEE’s factors. In Equation (4), EG, TP, UL, ES, HC, TL serve as the explanatory variable, and the CIGTFEE measured by Super-EBM serves as the explained variable.

(4) $$\begin{array}{l} CITFEE_{i,t}=\alpha_{i}+\phi_{t}+\beta_{1}EG_{i,t}+\beta_{2}TP_{i,t}+\beta_{3}UL_{i,t}+\;\beta_{4}ES_{i,t}\;+\beta_{5}HC_{i,t}\;+\beta_{6}TL_{i,t}\\ \;\;\;\;\;\;\;\;\;\;\;\;\;\;\;\;\;\;\;\;+\delta \sum_{j}W_{ij}(CITFEE_{i,t})+\mu_{i,t}\\ \mu_{i,t}=\lambda \sum_{j}W_{ij}\times u_{i,t}+\varepsilon_{i,t} \end{array}$$

where δ are the spatial autoregressive parameters, λ is the spatial error parameter, and the spatial econometric model can be expressed as the values of both. If δ = 0, and λ ≠ 0, then the form of spatial error model (SEM) should take; If λ = 0, and δ ≠ 0, then the form of spatial auto-regressive model (SAR) should take; if λ = δ = 0, then this model is a common model without spatial effects; αi, $\phi_{t}$ are spatially-fixed and time-fixed effects of the residual terms of the model, respectively; CIGTFEE, as an abbreviation for total factor energy efficiency of the construction industry, is the independent variable of the model. Economic growth, technological progress, urbanization level, energy structure, human capital, technology and equipment level can be respectively expressed by EG, TP, UL, ES, HC, TL; $\beta_{1}$, $\beta_{2}$, $\beta_{3}$, $\beta_{4}$, $\beta_{5}$, $\beta_{6}$ are coefficients of corresponding variables, that is, the regression coefficient; i and t corresponding to the area and time respectively; μ is the residual term; ε is a random error.

#### 4.3.1. Spatial Correlation Test on Residual Terms

Based on Equation (4)., the regression analyses were conducted using the OLS, and further test whether spatial autocorrelation is present in the residuals of regression models estimated. The results of test are shown in Table 4. To further demonstrate the necessity of controlling the fixed effects to improve the strength of the model interpretation, the estimation and testing results from the non-fixed effects model, space fixed effects model, time fixed effects model, and two-way fixed effects model are also presented in Table 4. The best-fit model could be distinguished by comparing four model parameters.

As shown in Table 4, the R-squared value of the time-fixed effect model is 0.758, which is significantly higher than that of non-fixed effects model (0.511), space fixed effects model (0.334) and two-way fixed effects model (0.178), indicating that the goodness-of-fit of the time-fixed effect model was best. In addition, the DW values of non-fixed effects model, space fixed effects model, time fixed effects model, and two-way fixed effects model were 1.529, 1.822, 2.072, and 1.885, respectively. Likewise, time fixed effects model achieved the largest DW value. Through the comparison of the above two parameters, it is shown that the key parameters of time fixed effects model are better than those in other models. That is, time fixed effects model has the best explanatory power in this paper. Thus, the time fixed effects model was adopted for subsequent empirical analysis.

At the same time, the test results of spatial autocorrelation of model residual terms are also given in the lower part of Table 4. In the time-fixed effects model, the LM-lag value of 2.844 passed the significance test with *p*-values < 0.1, while the LM-err value of 2.340 also passed the 10% significant level test, which fully shows that the residual term of time fixed effects model has significant spatial autocorrelation. The premise of the establishment of the traditional model is that the residual terms of the model are independent, and there is no connection. However, the residual terms of the model have spatial autocorrelation, using the least squares method can result in bias in the estimation results. Therefore, it is necessary to re-estimate the model by using the spatial econometric method. In addition, because the value of LM-lag is larger than LM-err, compared with the spatial error model, this means SAR has a better explanation than SEM. Thus, SAR is a better choice for this paper.

#### 4.3.2. Analysis on Driving Factors of the CIGTFEE in YREB

The aforementioned study confirms the significant geographical correlations across YREB provincial CIGTFEEs. Therefore, a spatial econometric model are adopted to overcome the biased estimation results brought by traditional non-spatial econometric methods. As shown in Table 5, the spatial autoregressive term W*dep. var and the spatial error term Spat. aut., estimated by Maximum Likelihood Estimate (MLE), were 0.101 and −0.269, and have passed the significance test at 10% and 1% level, respectively. This further demonstrated the rationality of the spatial econometric model. Compared with the estimation results of the OLS, the R-squared of the spatial econometric model was much higher, indicating that the spatial econometric model has a better fit than the OLS. At the same time, the *t*-test values of most independent variables in the spatial econometric model were improved on the basis of the common model, which also indicated that the estimation results of the model have been further optimized. In addition, by comparing the R-squared values of SAR and SEM, it can be seen that the former is larger than the latter, indicating that SAR had stronger explanatory power. Therefore, the estimation results of SAR should be taken to interpret the coefficients of variables.

Economic growth (EG) has a considerable positive impact on CIGTFEE at a significant level of 1%, indicating that an increase in regional per capita GDP is favourable to enhancing CIGTFEE. The more developed the economy, the more suitable support for the construction industry’s expansion, in terms of technology, talent, market, and capital, will be accessible. The more mature the regional construction industry’s development, the higher the level of energy use in the building industry will be. The result also explains why CIGTFEE in the downstream area of the economically developed Yangtze River Economic Belt is higher than that in the upstream area.

Contrary to expectations, the computed coefficient of technological progress (TP) was negative, failing every significance tests. This finding is directly tied to the current direction of technology R&D activity. According to the findings of Acemoglu, D., original technology research and development includes both clean technology and polluted technology [37]. If the firm is initially engaged in polluting technology, then technological research and development operations will simply increase pollutant emissions without reducing them. Although construction enterprises’ R&D investment of YREB is gradually increasing, most construction enterprises prioritize the economic benefits of technology R&D over the ecological benefits, resulting in the emergence of more pollution-oriented technologies that are unfriendly to the environment and will exacerbate pollutant discharge.

As expected, Urbanization (UL) had a significant inhibitory effect on CIGTFEE, implying that the excessive pursuit of urbanization had led to the rapid expansion of the construction industry, resulting in extensive development of the construction industry, which further aggravated the construction industry’s energy consumption and pollution, and exerted downward pressure on energy conservation and emission reduction in the local construction industry. It is clear that the development of the construction industry is closely tied to the urbanization of the YREB. With the urbanization process speeding up, paying close attention to the construction industry’s resource consumption is a critical approach to successfully raising the industry’s green level, which helps to progressively realize an efficient and energy-saving construction industry.

The estimation coefficient of Energy structure (ES) was positive, passing the significant test at 1% level, indicating that the higher the proportion of electric energy consumption to the total energy consumption in the construction industry, the greater the energy efficiency. That is, electricity, as a type of clean and efficient energy, can effectively reduce the carbon dioxide emissions of the construction industry with its wide application in construction, thus playing a positive role in promoting the greening process of the construction industry and improving CIGTFEE.

As expected, Human capital (HC) had a positive influence on the CIGTFEE at a significant level of 1%. The labor force is an essential input factor to support the development of the construction industry, and how much advanced technology is applied is determined by the education level of the labor force. Modern construction technology and equipment machinery have been applied with the gradual improvement of the labor force’s education level in the construction industry, which undoubtedly improves the overall labor productivity of the construction industry, reduces energy consumption, and produces greater economic benefits.

The effect of Technology and equipment level (TL) on CIGTFEE was positive, but not statistically significant, which was consistent with the research conclusion of Wang [38]. The possible reason is that, although improving the technical equipment rate of the construction industry is conducive to the effective utilization of resources and the reduction of energy consumption, the equipment of construction enterprises in some areas of the YREB has some issues, such as low technical degree, serious aging, high energy consumption and low production efficiency.

## 5. Conclusions and Policy Implication

### 5.1. Conclusions

Improving energy efficiency has become the key solution to deal with the energy-economic-environmental challenges. This paper develops and constructs an evaluation index system to evaluate the CIGTFEEs of 11 YREB provinces from 2003 to 2018 using the Super-EBM model considering undesirable outputs. The spatiotemporal dynamics analysis and spatial correlations analysis of the CIGTFEEs are performed to master the geographical and temporal distribution characteristics. In addition, using a spatial econometric model, this paper empirically tests the effects of six potentially important factors on CIGTFEE. The primary conclusions are as follows:

(1) During the sample period, only Jiangsu, Zhejiang, and Shanghai’s CIGTFEE are in a state of effective, while other provinces fail to achieve the effective state, and there is room for improvement.
(2) In terms of temporal evolution, the CIGTFEE during the research periods revealed an overall growing tendency. The CIGTFEE in the YREB’s upstream, midstream, and downstream followed different trajectories. Prior to 2006, the CIGTFEE in the downstream grew dramatically, then stayed rather steady. In the midstream, they had an “M” shape with “two peaks and one valley”. The inverted “U” curve was the trend of the upstream.
(3) Global Moran’s I testify the significant spatial correlations between provincial CIGTFEEs. The distribution of CIGTFEE in YREB generally exhibits a ladder-shaped change trend, with the regional average CIGTFEE from high to low levels was as follows: Downstream, Midstream and Upstream. Overall, the provinces in the downstream are clearly polarized with those in the upstream.
(4) The results of spatial econometric model demonstrated that the CIGTFEE is significantly promoted by EG, ES and HC, suppressed by UL, yet the impact of TP and TL is not significant.

### 5.2. Policy Implication

Based on the above analysis, some policies should be put forward to boost YREB’s CIGTFEE. To begin with, the environmental challenge should be included in the research framework of energy efficiency in the context of green development. Otherwise, the estimate of efficiency may be skewed and misleading to policymakers. Next, owing to the clear regional differences in CIGTFEEs, the government should formulate differentiated support policies to guide different regions to accelerate energy conservation and emission reduction of construction industry in light of its actual situation. On the one hand, the government should support the downstream in its efforts to advance green industrial transformation, improve environmental quality, achieve high-quality industrial development, and promote energy efficiency of construction industry. On the other hand, the government should increase its financial, human, and technological assistance for industrial energy conservation and emission reduction in the upstream and midstream. Furthermore, the government should concentrate on transforming conventional high-energy, high-emission sectors, facilitating the shift from extensive to intensive growth. Finally, given the clear spatial correlation between provincial CIGTFEEs, spatial layout is a key factor that must be taken into consideration in the planning of the construction industry in the YREB. A cross-regional cooperation and exchange mechanism should be built to promote the free flow of talents, technology, capital, and other factors across regions, particularly the transfer of advanced construction technology, equipment, and management models from the advanced downstream to the backward midstream and upstream, allowing the latter to upgrade and transform their construction industryand then decrease the regional gap.

The results of the spatial econometric model demonstrate that four factors have a significant impact on CIGTFEE. Accordingly, provinces in YREB must develop corresponding active policies and strategies to improve the energy utilization level of the construction industry. (1) Empirical results show that EG has a significant positive relationship with CIGTFEE. Taking advantage of YREB’s high-quality economic development, there is a need to accelerate the shifting of the traditional extensive development path of the “labor-intensive and energy-intensive” construction industry, actively promote construction industry transformation and upgrading, and realize the transformation of construction industry development from factor-driven to innovation-driven. (2) The improvement of UL hinders CIGTFEE growth. Hence, each province should actively pursue new urbanism development that is intensive, intelligent, green, and low-carbon to provide support for the coordinated growth of new urbanization construction and the construction industry. (3) ES optimization can greatly improve CIGTFEE. Each region should vigorously implement a green energy substitution strategy in the construction industry, encouraging the use of clean energy and green building materials while gradually reducing reliance on traditional fossil fuels like coal and oil. (4) HC has a promoting influence on CIGTFEE. During the critical moment of transformation and upgrading of the construction industry, YREB’s fundamental shortcoming is a small pool of high-tech expertise. It is critical to cultivate interdisciplinary innovation talent, establish professional groups for the industrial chain, and give more sophisticated talent for transformation and upgrade of the construction industry.

## Figures and Tables

**Table 1:** Measurement index of CIGTFEE of the construction industry.

Input/Output	Variable	Meaning (Units)
Inputs	Energy input	Comprehensive energy consumption of the construction industry (1000 t)
	Labor input	The number of employees of industrial enterprises in the region (1000 people)
	Capital input	The actual net construction fixed assets in the region with 2000 as the base period (CNY 100 million yuan)
Desirable output	Economic output	The actual total output of construction in the region with 2003 as the base period (CNY 100 million yuan)
Undesirable output	Carbon dioxide emissions	The carbon dioxide emissions of construction in the region (10,000 t)

**Table 2:** Details of the CIGTFEEs of 11 provinces in YREB from 2003 to 2018.

	2003	2004	2005	2006	2007	2008	2009	2010	2011	2012	2013	2014	2015	2016	2017	2018	Mean
Shanghai	1.029	1.027	1.052	1.069	1.072	1.110	1.097	1.103	1.097	1.045	1.024	1.015	1.033	1.015	1.004	0.862	1.041
Jiangsu	1.009	1.015	1.244	1.133	1.156	1.186	1.162	1.169	1.340	1.164	1.174	1.163	1.276	1.337	1.398	1.377	1.206
Zhejiang	1.045	1.053	1.075	1.067	1.051	1.055	1.050	1.032	1.075	1.034	1.034	1.043	1.051	1.099	1.106	1.102	1.061
Anhui	0.607	0.619	0.762	0.772	0.737	0.732	0.728	0.736	0.754	0.795	0.807	0.837	0.852	0.784	0.776	0.734	0.752
Jiangxi	0.787	0.820	1.128	0.878	0.840	0.834	0.876	1.008	0.763	1.018	1.014	1.000	0.980	0.847	0.809	0.857	0.904
Hubei	0.623	0.675	0.763	0.802	0.799	0.803	0.821	0.841	0.775	1.053	1.070	1.074	1.057	1.057	1.070	1.087	0.898
Hunan	0.867	0.679	0.754	0.778	0.795	0.814	0.809	0.811	0.821	0.794	0.804	0.812	0.809	0.787	0.765	0.759	0.791
Chongqing	0.664	0.599	0.734	0.705	0.695	0.704	0.736	0.731	0.715	0.796	0.855	0.844	0.832	0.805	0.767	0.787	0.748
Sichuan	0.562	0.558	0.653	0.696	0.694	0.705	0.729	0.673	0.665	0.790	0.835	0.803	0.806	0.772	0.725	0.690	0.710
Guizhou	0.566	0.592	0.679	0.703	0.695	0.661	0.703	0.715	0.740	0.821	0.810	0.750	0.806	0.701	0.703	0.651	0.706
Yunnan	0.546	0.577	0.664	0.666	0.660	0.654	0.705	0.709	0.686	0.741	0.761	0.734	0.743	0.679	0.681	0.678	0.680

**Table 3:** Spatial autocorrelation coefficient of the CIGTFEE of YREB in four periods.

Periods	2003–2006	2007–2010	2011–2014	2015–2018
Moran’s I	0.642	0.618	0.449	0.309
*p*-value	0.005	0.005	0.013	0.032
Z-value	3.523	3.580	2.767	2.241

**Table 4:** The estimation and test results of different fixed-effects models.

Variables	Non-Fixed Effects Model	Space Fixed Effects Model	Time Fixed Effects Model	Two-Way Fixed Effects Model
EG	0.194 *** (3.220)	0.094 (0.994)	1.273 *** (13.849)	0.127 (0.709)
TP	0.007 (0.240)	−0.048 ** (−2.111)	−0.001 (−0.043)	−0.0027 (−0.078)
UL	0.532 *** (2.598)	0.655 * (1.836)	−1.472 *** (−7.199)	1.286 *** (3.534)
ES	0.108 (1.003)	−0.009 (−0.129)	0.475 *** (5.808)	0.170 ** (2.372)
HC	−0.017 (−0.628)	−0.015 (−0.683)	0.081 *** (3.909)	0.020 (0.747)
TL	−0.026 (−0.977)	0.006 (0.322)	0.017 (0.882)	−0.005 (−0.264)
R-squared	0.511	0.334	0.758	0.178
DW	1.529	1.822	2.072	1.885
LM-lag	9.080 ***	4.707 **	2.844 *	1.052
Robust LM-lag	14.660 ***	8.979 ***	20.186 ***	5.067 **
LM-err	4.106 **	7.195 ***	2.340 *	0.355
Robust LM-err	9.686 ***	11.467 ***	19.682 ***	4.369 **

Note: The *t*-statistic is bracketed; ‘*’, ‘**’, and ‘***’ are significance levels of 10%, 5%, and 1%, respectively; model estimation and spatial auto-correlation test were conducted on MATLAB 7.12.

**Table 5:** The estimation and test results of spatial econometric model (time fixed effects model).

Variables	SAR	SEM
EG	1.263 *** (14.013)	1.273 *** (13.592)
TP	−0.006 (−0.311)	0.023 (1.237)
UL	−1.490 *** (−7.430)	−1.585 *** (−7.424)
ES	0.459 *** (5.727)	0.470 *** (5.676)
HC	0.078 *** (3.826)	0.091 *** (4.563)
TL	0.018 (0.997)	0.0010 (0.516)
W*dep. var	0.101 * (1.537)	
Spat. aut.		−0.269 *** (−2.609)
R-squared	0.781	0.774

Note: The *t*-statistic is bracketed; * and *** are significance levels of 10% and 1%, respectively.

## Data Availability

The data used to support the findings of this study are available from the corresponding author upon request (e-mail: zhaona1997@2020.cqut.edu.cn).
